# Global Patterns of Visual Disability and Low Vision Aid Prescription: A Systematic Review

**DOI:** 10.7759/cureus.113756

**Published:** 2026-07-31

**Authors:** Vidhya Verma, Saroj Gupta, Priti Singh, Samendra Karkhur, Pratyush Ranjan Behera

**Affiliations:** 1 Ophthalmology, All India Institute of Medical Sciences, Bhopal, Bhopal, IND; 2 General Practice, All India Institute of Medical Sciences, Bhubaneswar, Bhubaneswar, IND

**Keywords:** assistive visual devices, low vision, low vision aids, low vision rehabilitation, visual disability, visual impairment

## Abstract

Visual disability remains a major global public health challenge, particularly among individuals with irreversible ocular diseases where medical or surgical treatment cannot restore functional vision. Low-vision rehabilitation (LVR), including the prescription of low-vision aids (LVAs), plays a critical role in optimizing residual vision and improving quality of life; however, patterns of visual disability and LVA utilization vary widely across populations and healthcare settings. This systematic review was conducted in accordance with PRISMA 2020 guidelines to evaluate patterns of visual disability and the prescription, utilisation, and functional outcomes of low-vision aids across diverse patient populations. Electronic databases, including PubMed, Scopus, EMBASE, and the Cochrane Library, along with Google Scholar and citation tracking, were searched for studies published between 2000 and 2025. Observational studies, randomized or crossover trials, service audits, and rehabilitation reports assessing low-vision rehabilitation or assistive devices were included.

Of 975 records identified, 19 studies met the inclusion criteria. Retinal diseases such as age-related macular degeneration, diabetic maculopathy, and inherited retinal dystrophies were the predominant causes of visual disability in adults, whereas congenital and developmental conditions were more common in pediatric populations. Optical LVAs, particularly high-add spectacles and magnifiers, were the most frequently prescribed devices, while electronic and wearable aids demonstrated superior task-specific performance but lower real-world uptake due to cost and usability barriers. Telerehabilitation showed functional outcomes comparable to in-clinic training. Overall, low-vision aids significantly improve functional outcomes across a wide spectrum of visual disabilities, and a multimodal, patient-centered rehabilitation model integrating optical, electronic, and tele-rehabilitation approaches is essential to enhance accessibility, service reach, and effectiveness.

## Introduction and background

Visual disability is a major public health concern that significantly limits daily functioning, academic and occupational performance, mobility, and overall quality of life. The global pattern of visual impairment reflects a dual burden: age-related ocular diseases - such as age-related macular degeneration, diabetic retinopathy, and glaucoma - predominate in adults and the elderly, while congenital and childhood causes, including hereditary retinal dystrophies, optic nerve anomalies, and developmental disorders, constitute a substantial proportion of early-onset disability [1,2]. When medical or surgical interventions are insufficient to restore functional vision, low-vision rehabilitation (LVR) becomes essential for optimising residual visual capacity. LVR becomes essential for optimizing residual visual capacity, as emphasized in World Health Organization frameworks and established rehabilitation guidelines [3,4].

Visual disability is generally defined as a clinically significant reduction in visual function that persists despite optimal refractive correction or available therapeutic interventions and restricts an individual’s ability to perform routine activities. Epidemiologic definitions commonly rely on World Health Organisation criteria, where visual disability encompasses presenting visual acuity worse than 6/18 in the better eye, while blindness is additionally defined by severe visual field constriction (<10°) [3]. Functional definitions used in rehabilitation settings emphasize task-specific difficulties and the need for assistive devices [3,4]. In population-based research, classifications proposed by the Vision Loss Expert Group are widely used to quantify moderate-to-severe vision impairment and blindness [1].

Low vision rehabilitation encompasses a multidisciplinary approach involving ophthalmologists, optometrists, low vision specialists, occupational therapists, orientation and mobility instructors, rehabilitation professionals, and caregivers. Appropriate assessment, selection of optical and electronic low vision devices, patient training, and regular follow-up are essential to optimise device utilisation, improve reading and mobility, support safe daily activities including driving where appropriate, and enhance overall vision-related quality of life.

Recent global estimates indicate that approximately 295 million individuals live with moderate-to-severe visual impairment, and 43 million are blind [5]. Furthermore, the WHO reports that at least 1 billion people have vision impairment that remains unaddressed, many of whom would benefit more from rehabilitation than curative treatment alone [3]. Despite this considerable burden, the availability and accessibility of LVR services remain limited worldwide, particularly in low- and middle-income regions where trained personnel, specialised clinics, and affordable devices are scarce [5]. These disparities underscore the need for systematic assessment of visual disability patterns and the utilization of LVR services.

The present systematic review aims to evaluate the patterns of visual disability and the prescription of low-vision aids, with a focus on identifying commonly used devices, rehabilitation strategies, and their functional outcomes. Additionally, the review seeks to assess awareness and utilization of low-vision aids among visually impaired individuals to inform service delivery models and guide improvements in low-vision care.

## Review

Methods

Protocol and Reporting Standards

This systematic review was conducted and reported in accordance with the Preferred Reporting Items for Systematic Reviews and Meta-Analyses (PRISMA) guidelines [6]. The protocol for this review was prospectively registered with Prospero ID: CRD42023469395, and all steps adhered to the recommended standards for transparent reporting of systematic reviews.

Search Strategy

Literature search strategy: A comprehensive electronic literature search was conducted across PubMed, EMBASE, Scopus, the Cochrane Library, and Google Scholar to identify relevant studies published between January 1, 2000, and October 31, 2025. Additional records were identified through manual citation tracking of included articles and relevant reviews. The search strategy combined controlled vocabulary (MeSH terms) and free-text keywords, including low vision, visual impairment, visual disability, low vision aids, optical devices, electronic magnifiers, and visual rehabilitation. Boolean operators were applied as follows: (“low vision” OR “visual impairment” OR “visual disability”) AND (“low vision aids” OR “optical devices” OR “electronic magnifiers”). Searches were restricted to human studies published in English. For PubMed, location-specific terms were additionally used to identify Indian studies. The complete search strategy was adapted appropriately for each database.

Eligibility Criteria

Inclusion criteria: Studies were included if they met the following criteria:

They involved individuals with visual disability or low vision, as defined by the World Health Organisation (WHO) or equivalent functional criteria, and included both adult and paediatric populations [3]. Eligible study designs comprised observational studies, including cross-sectional, retrospective and prospective audits, cohort, case-control and ambispective studies, as well as randomised controlled trials (RCTs), crossover trials, economic evaluations, and rehabilitation service reports. Studies evaluating the prescription, assessment, or utilisation of low-vision aids - including optical, electronic, or digital devices - or visual skills training and assistive technologies were considered. Outcomes of interest included the aetiology of visual disability, patterns of low-vision aid prescription, device uptake and adherence, functional outcomes (such as near-vision improvement and visual ability scores), patient-reported outcomes (quality of life, usability, satisfaction), and economic or cost-related outcomes. Eligible conditions included irreversible causes of visual impairment, such as inherited retinal dystrophies, glaucomatous optic neuropathy, and age-related macular degeneration.

Exclusion criteria: Studies were excluded if they were case reports, narrative reviews, editorials, or commentaries, or if only an abstract was available and the full text could not be accessed. Studies that did not report rehabilitation- or device-related outcomes, those focused exclusively on reversible causes of visual impairment (such as cataract surgery), and laboratory-based, animal, or non-clinical experimental studies were also excluded.

Study Selection

All identified records were imported into Covidence software for deduplication and screening. Two independent reviewers screened titles and abstracts, followed by a full-text review of potentially eligible studies. Discrepancies were resolved by discussion until consensus was reached. The study selection process is illustrated in the PRISMA flow diagram.

Data Extraction and Quality Assessment

Two reviewers (V.V. and P.S.) independently screened titles and abstracts for relevance. Full-text articles of all potentially eligible studies were retrieved and assessed in detail. Any disagreements were resolved through discussion and consensus with senior reviewers (S.G. and S.K.). Data extraction was performed independently using a predesigned data extraction form, and extracted information was tabulated as “Characteristics of Included Studies.”

Extracted data included study characteristics (first author, year of publication, study design, country, and clinical setting), population details (sample size, age distribution, and underlying diagnosis or aetiology of visual impairment), and intervention or exposure characteristics (type of low-vision aid prescribed, training modality, or rehabilitation programme). Outcome measures included functional outcomes (such as near-vision improvement and reading speed), patient-reported outcomes (quality of life, usability, and satisfaction), adherence or compliance data, and economic or cost-related outcomes where reported. Follow-up duration, key findings, and effect estimates (means with standard deviations, proportions, and confidence intervals) were recorded where available.

Missing, unclear, or inconsistent numerical data were documented and addressed qualitatively in the Discussion section. Risk of bias assessment was performed using tools appropriate to the study design. Randomised and crossover trials were evaluated using the Cochrane Risk of Bias 2 (RoB 2) tool, assessing domains related to randomisation, allocation concealment, blinding feasibility, attrition, and selective outcome reporting. Non-randomised studies were assessed using an adapted ROBINS-I, focusing on participant selection, comparability of study groups, and outcome assessment. Economic evaluations were reviewed for the clarity of costing assumptions, analytic perspective, completeness of cost data, and transparency of reporting.

Risk of bias was summarised qualitatively. Observational studies were examined primarily for selection bias, confounding, and incomplete reporting, while randomised trials were assessed for allocation, performance, and detection biases, particularly those inherent to device-based interventions. The overall certainty of evidence across outcomes was evaluated using the GRADE approach [7].

Owing to substantial clinical and methodological heterogeneity among the included studies, findings were synthesized narratively by grouping studies according to intervention type and outcomes, with conflicting evidence interpreted in the context of study quality and design. Missing data were reported as available without imputation, and no deviations from the registered PROSPERO protocol occurred during the review.

Data Synthesis

Given the substantial heterogeneity in study populations, intervention types, low-vision devices, outcome measures, and follow-up durations, a narrative synthesis was undertaken. Studies were grouped according to population and diagnostic category (adults with retinal or optic nerve disease, children with congenital or developmental visual impairment, and mixed cohorts), type of device (optical, electronic, or digital/tablet-based), and outcome domain (patterns of low-vision aid prescription, functional performance, quality of life, acceptability, and cost).

Tabulated summaries were used to present ranges of aetiologies, device utilisation, and functional outcomes across studies. A quantitative meta-analysis was planned if at least three studies reported comparable numerical outcomes; however, pooled analyses were not feasible due to inconsistent reporting formats, variable follow-up periods, and substantial clinical and methodological heterogeneity. This review was based exclusively on published data, and no primary patient-level data were collected. Extracted and analysed data are available from the authors upon reasonable request.

Results

The systematic search identified 953 records through database searches (PubMed, Scopus, Embase, and the Cochrane Library) and an additional 22 records through Google Scholar and manual citation tracking. After removal of 200 duplicate records, 753 records remained and were screened at the title and abstract level. Of these, 661 records were excluded as irrelevant to low-vision rehabilitation or assistive device use.

**Figure 1:**
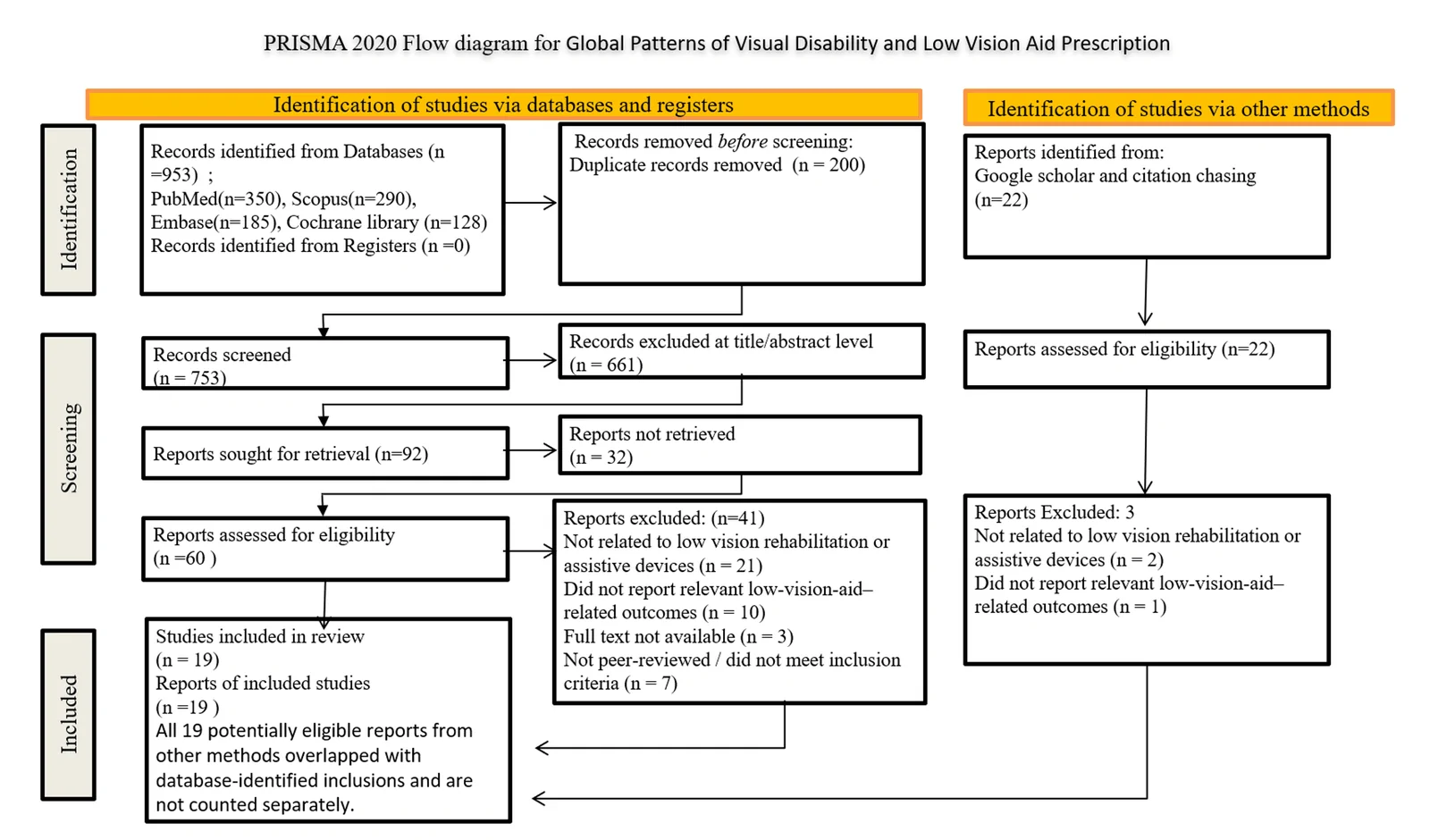
PRISMA flow chart showing the process of selecting articles/studies for the systematic review. PRISMA: Preferred Reporting Items for Systematic Reviews and Meta-Analyses

A total of 92 reports were sought for full-text retrieval, of which 32 reports could not be retrieved. Consequently, 60 full-text reports were assessed for eligibility. Among these, 41 reports were excluded for the following reasons: not related to low-vision rehabilitation or assistive devices (n = 21), failure to report relevant low-vision-aid-related outcomes (n = 10), full text not available (n = 3), and not peer-reviewed or not meeting inclusion criteria (n = 7). In parallel, 22 reports identified through other methods were assessed for eligibility, of which three reports were excluded due to lack of relevance to low-vision rehabilitation (n = 2) or absence of relevant low-vision-aid outcomes (n = 1). Overall, 19 studies met the inclusion criteria and were included in the final systematic review. The study selection process is summarised in Figure 1.

Study Characteristics

The 19 studies included in the systematic review represented a broad range of study designs, including randomized controlled trials, crossover trials, observational studies, pre-post intervention evaluations, feasibility trials, and service audits. Sample sizes varied substantially, ranging from 30 to 3,470 participants. Study populations included adults with retinal and optic nerve disorders, paediatric patients with congenital or developmental visual impairment, and mixed cohorts of low-vision clinic attendees, reflecting real-world rehabilitation settings.

**Figure 2:**
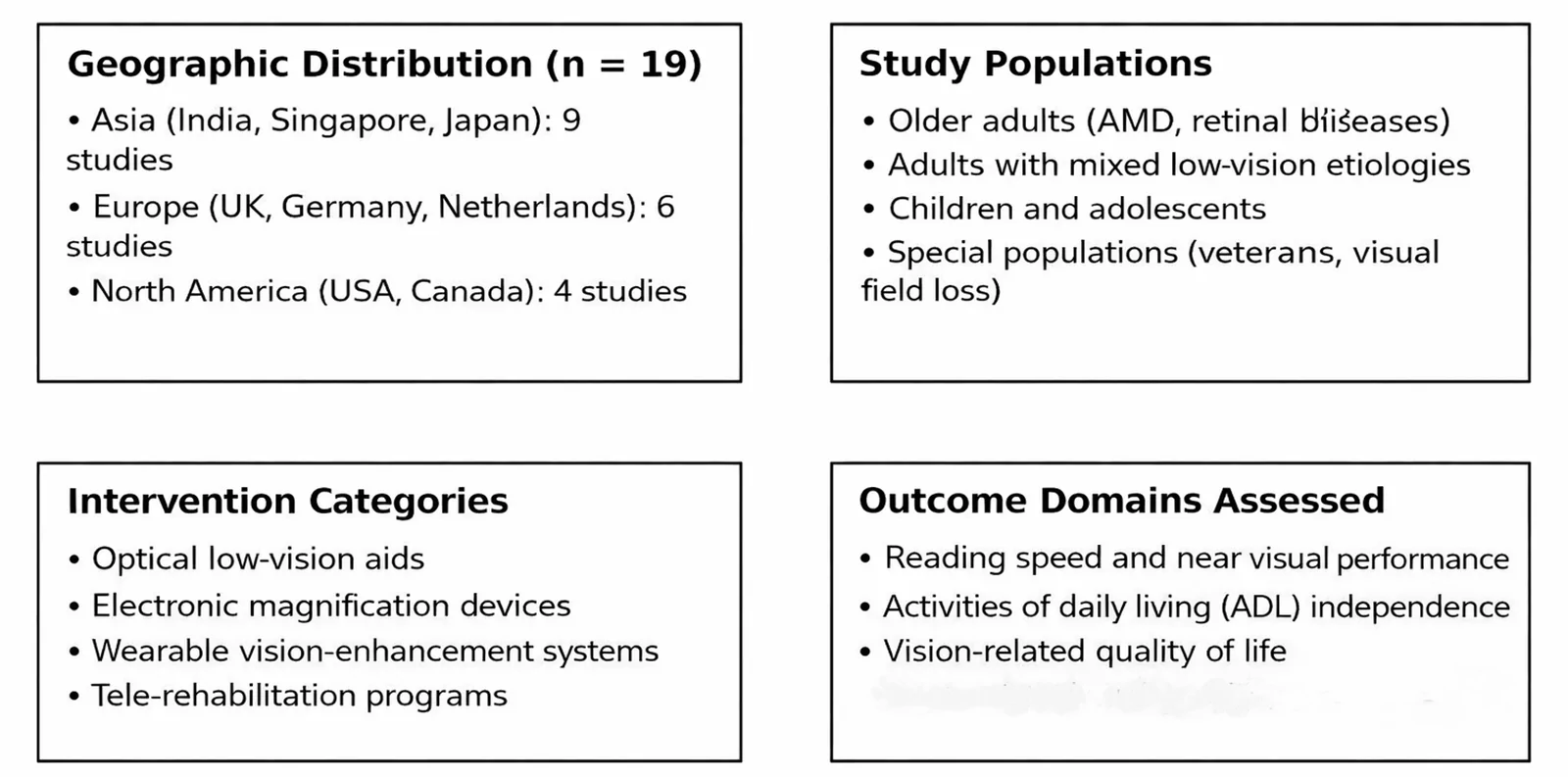
Summary of Low Vision Rehabilitation Evidence From Included Studies The figure summarizes characteristics of included studies. Geographic distribution, study populations, intervention categories, and outcome domains are assessed.

The studies were conducted across multiple geographic regions, including India, the United Kingdom, the United States, Germany, Singapore, Canada, and Japan, highlighting considerable international and clinical diversity in low-vision rehabilitation practices (Figure 2).

A wide spectrum of low-vision aids (LVAs) and rehabilitation strategies was evaluated. Interventions included optical devices such as high-add spectacles, hand and stand magnifiers, spectacle-mounted magnifiers, and telescopic aids; electronic devices, including desktop and portable closed-circuit television (CCTV) systems, wearable electronic vision enhancement systems (wEVES) such as head-mounted displays (e.g., eSight), and portable electronic vision enhancement systems (p-EVES); and digital aids, comprising tablets, smartphones, and computer-based assistive technologies. Several studies also incorporated training and rehabilitation modalities, including clinic-based training for optical and electronic devices, telerehabilitation for follow-up and reinforcement, and structured self-management programmes aimed at improving device use and adherence. The key characteristics of the included studies - covering country and setting, study population, intervention and comparator details, and outcome measures - are summarised in Table 1.

**Table 1:** Characteristics of Low Vision Rehabilitation Studies AMD: Age-related macular degeneration; LVA: Low-vision aid; LVQOL: Low Vision Quality of Life; NEI VFQ-25: National Eye Institute Visual Function Questionnaire-25; ADL: Activities of daily living; CCTV: Closed-circuit television; VRQoL: Vision-related quality of life; QALY: Quality-Adjusted Life Year; HRQoL: Health-Related Quality of Life

Author (First)	Year	Country / Setting	Sample Size (N)	Age Group (Mean ± SD)	Population	Intervention Type	Comparator	Outcome Measures
Do [8]	2014	India (Aravind Eye Hospital, rural South India)	55 (44 completed)	Not specified	Low-vision patients referred to clinic	Low-vision examination + LVAs	Normal-vision controls	LVQOL (mobility, adjustment, reading, ADL); QoL improvement
Singh [9]	2023	India	30	68 ± 10 years	Elderly patients with AMD (dry & wet)	LVAs (spectacles, magnifiers, prisms)	Pre-LVA baseline	Near visual acuity, reading speed (wpm), ADL questionnaire
Oeverhaus [10]	2020	Germany	34	Not specified	Patients with corneal disease	LVAs (optical, electronic, CCTV)	Cross-over between devices	Reading speed (IReST), corneal densitometry
Crossland [11]	2017	India & UK	40	10–18 years	Children and young people with low vision	iPad vs optical LVAs	Standard low-vision care	Feasibility, acceptability, VRQoL, educational outcomes
Gothwal [12]	2018	India & UK	40	10–18 years	Children and young people with low vision	Tablet computers (RCT)	Standard low-vision care	Recruitment, retention, device usage, accessibility
Miller [13]	2025	UK	34	80.2 ± 6 years	Patients with AMD	Wearable electronic vision enhancement system (wEVES)	Cross-over trial	Qualitative outcomes: usability, barriers (thematic analysis)
Bittner [14]	2022	USA (multi-phase)	45 (10 + 11 + 24)	Not specified	Adults with visual impairment	Telerehabilitation for magnifier use	In-office training	Acceptance, satisfaction, magnifier use
Bray [15]	2017	UK	82	Not specified	Optical LVA users	Portable electronic vision enhancement systems (p-EVES)	Optical LVAs only	Cost-effectiveness, QoL, QALYs, near-vision function
Visser [16]	2020	Netherlands	190	Not specified	Patients with AMD	E-Scoop spectacle-mounted lens	Standard optical correction	QoL (NEI VFQ-25), visual acuity, contrast sensitivity
Bittner [17]	2024	USA (multicenter)	61 (47 completed)	Not specified	Adults with low vision	Telerehabilitation vs in-office magnifier training	Randomized groups	Reading ability (Activity Inventory; Rasch logits)
Tey [18]	2019	Singapore	165 (128 completed)	Not specified	Low-vision patients	“Living Successfully with Low Vision” self-management program	Usual care (LVA training only)	VRQoL (IVI), HRQoL, mental health, self-efficacy
Taylor [19]	2017	UK	100 (82 completed)	Not specified	Experienced optical aid users	Portable electronic magnifiers (p-EVES)	Optical LVAs only	Reading speed, task independence, near-vision activities
Lorenzini [20]	2021	Canada	57	Not specified	eSight head-mounted display users	Telerehabilitation	Manufacturer self-training	Feasibility, acceptability, device-use behaviour
Nguyen [21]	2022	USA	3,470 exam records	Not applicable	Low-vision clinic patients	Longitudinal device assessments	Historical trends	Case history themes, device assessment patterns
Senjam [22]	2019	India (Delhi tertiary centre)	85	Mostly young patients	Young patients with visual disability	Assistive technology awareness survey	None	Awareness, utilisation, perceived barriers
Acton [23]	2016	UK	Protocol (planned N not specified)	Not specified	Low-vision patients	Rehabilitation worker input	Usual care	Visual function, psychosocial outcomes
Stelmack [24]	2008	USA (VA centres)	126	Not specified	Veterans with macular disease	VA Low Vision Intervention Trial (LOVIT)	Waiting-list control	VA LV VFQ-48 (reading, mobility, information processing, motor skills)
Peterson [25]	2003	UK	70	Not specified	Visually impaired patients	Electronic vision enhancement systems (EVES)	Optical magnifiers	Reading speed, map tracking, medicine-label identification
Mawatari [26]	2024	Japan	Randomized crossover trial (N not fully reported)	Not specified	Adults with concentric peripheral visual field loss	Wearable night-vision aid	Cross-over comparison	Functional efficacy in peripheral visual field loss

Populations and causes of visual disability

Adult and Paediatric Populations

Across adult populations, retinal disorders emerged as the most frequently reported causes of irreversible visual impairment. Age-related macular degeneration (AMD), encompassing both dry and wet forms, was the predominant diagnosis, followed by diabetic maculopathy and inherited retinal dystrophies such as retinitis pigmentosa. Other contributors to permanent visual disability included optic atrophy, advanced glaucomatous optic neuropathy, and corneal disease. Studies conducted at tertiary referral centres tended to overrepresent patients with inherited retinal dystrophies and advanced stages of disease, reflecting referral bias inherent to specialised low-vision rehabilitation (LVR) services.

In paediatric cohorts, congenital and developmental causes predominated. The most commonly reported aetiologies included congenital ocular anomalies such as anophthalmia and microphthalmia, albinism, cortical visual impairment, corneal opacities, and refractive amblyopia. These conditions often resulted in lifelong visual disability and necessitated early rehabilitation and educational support.

Quantitative estimates of causes of visual disability

Across population-based and clinic-based studies, cataract remained a major contributor to visual disability, accounting for approximately 21-48% of cases, followed by uncorrected refractive errors (18-36%). Diabetic retinopathy contributed to 5-12% of cases, while glaucoma accounted for 2-8%. Inherited retinal dystrophies constituted 5-28% of cases overall, with substantially higher proportions reported in tertiary LVR clinics, reflecting selective referral of complex and advanced cases.

Patterns of low-vision aid prescription

Near-vision aids were the most frequently prescribed low-vision devices across studies. These primarily included high-add spectacles, hand and stand magnifiers, and spectacle-mounted magnifiers. Distance-vision aids, such as monocular and spectacle-mounted telescopes, were prescribed less commonly. Electronic low-vision aids - including desktop and portable closed-circuit television (CCTV) systems and wearable vision-enhancement devices - consistently demonstrated superior reading performance, smaller achievable print sizes, and improved functional independence. However, their uptake remained limited owing to high cost, portability issues, and reduced familiarity with technology among some patient groups.

Digital aids, particularly tablet- and computer-based assistive technologies, were more frequently utilised in paediatric populations and educational rehabilitation settings, often within structured training or school-based programs.

Functional outcomes and acceptance

Low-vision rehabilitation was associated with consistent improvements in near-vision performance, reading speed, task independence, and activities of daily living across adult and paediatric populations. In children, rehabilitation interventions were additionally linked to improved educational participation and functional classroom performance. Simpler optical devices demonstrated the highest levels of acceptance and long-term adherence, likely due to their affordability, ease of use, and familiarity. In contrast, telescopic devices showed lower acceptance, commonly attributed to cosmetic concerns and handling difficulties.

Electronic magnifiers and portable electronic devices yielded meaningful improvements in functional outcomes, particularly for tasks requiring sustained reading or very small print sizes. Importantly, telerehabilitation-based interventions achieved functional outcomes comparable to traditional in-office training, supporting their feasibility as an alternative model of care in resource-limited or geographically underserved settings.

Barriers to utilisation and service delivery implications

Several recurring barriers to low-vision aid utilisation were identified across studies, including the high cost of electronic and advanced optical devices, limited awareness among patients and healthcare providers, shortages of trained low-vision rehabilitation personnel, cosmetic concerns - especially related to telescopes - and inadequate follow-up and training support for device use. Service delivery reports highlighted the need for function-based prescription approaches, stronger integration between community-level and tertiary rehabilitation services, multidisciplinary teams with specialised training, and structured follow-up programs to reinforce device utilisation. Telerehabilitation was consistently identified as a promising strategy to address workforce limitations and improve access to care, particularly where technological infrastructure and patient support were available.

Risk of bias assessment

Randomised controlled trials were assessed using the Cochrane Risk of Bias 2 tool, while nonrandomised studies were evaluated using ROBINS-I. Most RCTs demonstrated a low risk of bias in randomisation procedures and outcome measurement, as observed in studies such as Stelmack [24], Visser et al. [16], and Bittner et al. [17]. Some crossover trials raised concerns regarding period and carryover effects, and feasibility studies exhibited a higher risk due to attrition and an emphasis on acceptability rather than efficacy outcomes.

Nonrandomised studies commonly demonstrated serious risks of confounding and selection bias, particularly in pre-post designs and cross-sectional surveys. Although within-participant task-based designs helped reduce between-group confounding, they remained susceptible to learning and fatigue effects.

Tables 2-4 provide a detailed risk-of-bias assessment for all included studies.

**Table 2:** Risk of Bias Assessment of Randomized and Crossover Trials (Cochrane RoB 2) VA, LV VFQ-48: Veterans Affairs Low-Vision Visual Functioning Questionnaire-48; HRQoL: Health-Related Quality of Life

Study (Year)	Design	Randomization Process	Deviations from Intended Interventions	Missing Outcome Data	Measurement of Outcomes	Selection of Reported Results	Overall Risk of Bias
Stelmack 2008 [24]	RCT, masked outcome assessor	Low: centralized enrollment, clear arms	Low: structured program vs waitlist	Low: telephone follow-up, high completion	Low: validated VA, LV VFQ-48	Low: prespecified domains	Low
Taylor et al., 2017 [19]	Two-arm randomized crossover	Some concerns: period/carryover risk	Low: standardized device phases	Low: 82/100 completed	Low: reading speed, task performance	Low: defined outcomes	Some concerns
Bray et al., 2017 [15]	Economic evaluation alongside crossover RCT	Some concerns: same parent trial	Low: device addition vs control	Low	Low	Some concerns: model assumptions	Some concerns
Visser et al., 2020 [16]	RCT, open-label	Low: large sample, clear allocation	Low: optimized optical refraction	Low	Low: standardized VA, logCS	Low	Low
Tey et al., 2019 [18]	RCT	Low: parallel groups	Low	Some concerns: 22% non-completion	Low: IVI, HRQoL	Low	Some concerns
Lorenzini & Wittich, 2021 [20]	Randomized feasibility trial	Some concerns: feasibility focus	Some concerns: differential withdrawal	High: 35% withdrew	Some concerns: qualitative performance	Some concerns	High
Bittner et al., 2024 [17]	Multicenter RCT	Low: randomized, multilevel modeling	Low: standardized remote vs in-office training	Some concerns: 47/61 completed	Low: Rasch-logit reading ability	Low	Low
Oeverhaus et al., 2020 [10]	Randomized crossover	Some concerns: order and carryover effects	Low: within-day standardized testing	Low	Low: IReST reading speed	Low	Some concerns
Mawatari et al., 2024 [26]	Single-blind, 3-period crossover	Some concerns: small sample, carryover	Low: predefined lens and lighting	Low	Low: visual field angle measurement	Low	Some concerns
Gothwal et al., 2018 [12]	Pilot RCT	Low: 1:1 allocation (UK & India)	Low	Some concerns: 85–95% retention	Low: feasibility outcomes	Low	Some concerns
Crossland et al., 2017 [11]	RCT protocol	Low: protocolized methods	Low	Low	Low	Low	Low

**Table 3:** Risk of Bias Assessment of Non-Randomized Studies (ROBINS-I) LVQOL: Low Vision Quality of Life; AMD: Age-related macular degeneration; ADL: Activities of daily living

Study (Year)	Design	Confounding	Deviations from Intended Interventions	Missing Outcome Data	Measurement of Outcomes	Selection of Reported Results	Overall Risk of Bias
Do et al., 2014 [8]	Pre–post with controls	Serious: baseline group differences	Moderate: referral-based sampling	Some concerns: 44/55 completed	Moderate: LVQOL (telephone)	Moderate	Serious
Singh et al., 2023 [9]	Prospective pre–post	Serious: no control group	Low: consecutive AMD patients	Low	Moderate: reading speed, ADL questionnaire	Moderate	Serious
Nguyen et al., 2022 [21]	Retrospective records review	Serious: secular trends, demographics	Low: clinic-based population	Low	Moderate: automated data extraction	Moderate	Serious
Senjam et al., 2019 [22]	Cross-sectional survey	Serious: confounding in awareness/utilization	Low: first-visit VR clinic patients	Low	Moderate: structured survey tool	Moderate	Serious
Peterson et al., 2003 [25]	Quasi-experimental, within-patient tasks	Moderate: learning/fatigue effects	Low: sequential clinic recruitment	Low	Low: task-based measures	Low	Moderate

**Table 4:** Risk of Bias Assessment of Qualitative / Other Studies

Study (Year)	Design	Confounding	Deviations from Intended Interventions	Missing Outcome Data	Measurement of Outcomes	Selection of Reported Results	Overall Risk of Bias
Miller et al., 2025 [13]	Qualitative study	Not applicable	Not applicable	Low	High: subjective interview data	High	High

Crossover RCTs commonly showed some concerns due to period or carryover effects. Feasibility trials had a higher risk of bias because of attrition and limited efficacy focus. Nonrandomized studies carried inherent confounding, though within-participant designs reduced some bias. Qualitative studies showed a high risk of bias due to subjectivity and a lack of controls.

Certainty of evidence (GRADE)

Outcomes across the included studies were evaluated using the GRADE framework for key functional and patient-centred domains, including reading ability, reading speed, accessible print size, vision-related quality of life, independence in activities of daily living, and contrast sensitivity. Overall, reading ability, typically expressed as logit scores, showed a moderate improvement following low-vision rehabilitation, with telerehabilitation interventions demonstrating functional outcomes comparable to in-office programmes; the certainty of evidence for this outcome was rated as moderate, limited by modest sample sizes and heterogeneity in intervention types. Reading speed exhibited small-to-moderate gains, particularly with the use of electronic magnifiers, with closed-circuit television systems producing the largest improvements; however, variability in device types and study contexts resulted in moderate-certainty evidence. Accessible print size consistently improved with electronic magnification, allowing users to read smaller print, with a moderate level of certainty.

Evidence for vision-related quality of life, assessed using instruments such as the NEI VFQ-25, IVI, and LVQOL, was mixed. While structured, programme-based rehabilitation demonstrated functional and psychosocial benefits, single-device interventions showed limited impact on quality-of-life outcomes, leading to a low certainty rating due to reliance on nonrandomized designs and inconsistent reporting. Independence in activities of daily living improved moderately with electronic magnifier use, supported by evidence of moderate certainty. Improvements in contrast sensitivity and distance visual acuity were statistically significant but clinically modest, and the certainty of evidence for these outcomes was also moderate. Finally, outcomes related to service delivery models, particularly telerehabilitation, indicated high acceptability and functional gains comparable to conventional in-office training, with an overall moderate certainty of evidence. Table 5 summarises the GRADE assessment for all outcomes.

**Table 5:** Summary of Outcomes and Certainty of Evidence (GRADE) VRQoL: Vision-related quality of life; NEI VFQ-25: National Eye Institute Visual Function Questionnaire-25; LVQOL: Low Vision Quality of Life; ADL: Activities of daily living

Outcome	Effect Direction and Magnitude	Evidence Base	Certainty (GRADE)	Key Reasons for Rating
Reading ability with magnifiers (logit change, Rasch)	Moderate improvement at 1–4 months; telerehabilitation comparable to in-office programmes	Multicentre RCTs	Moderate	Imprecision due to modest sample sizes; indirectness from heterogeneous devices and training protocols
Reading speed (words/min; near tasks)	Small-to-moderate improvement compared with optical-only aids; greatest gains with CCTV systems	RCTs and randomized crossover trials	Moderate	Inconsistency across device types and testing contexts; risk of bias from crossover period and carryover effects
Accessible / critical print size	Smaller print sizes achievable with electronic magnifiers compared with optical aids	Randomized crossover trials	Moderate	Risk of bias from crossover designs without full washout; indirectness due to laboratory-based task emphasis
Quality of life (VRQoL, NEI VFQ-25, IVI, LVQOL)	Mixed effects: programme-based rehabilitation improved functional domains, while some devices showed no meaningful QoL gains	RCTs and pre–post studies	Low	Inconsistency between device-only and service-based interventions; risk of bias in nonrandomized designs; imprecision
Independence / ADL performance	Greater task independence with addition of electronic magnifiers	Randomized crossover trials	Moderate	Risk of bias from crossover designs; indirectness due to short follow-up and variable task sets
Contrast sensitivity / distance acuity	Statistically significant but clinically small improvements with specific optical lenses	RCTs	Moderate	Imprecision from small effect sizes near measurement variability; indirectness with limited functional translation
Service delivery (telerehabilitation acceptability and equivalence)	High acceptability and functional gains comparable to in-office training	RCTs and feasibility RCTs	Moderate	Imprecision due to attrition in feasibility studies; indirectness related to technology and support requirements

Evidence synthesis across study clusters

Evidence synthesis across study clusters indicated that structured magnifier training, delivered either in-office or through telerehabilitation, consistently improved reading ability and near-vision task performance. Electronic magnifier crossover trials demonstrated superior reading speed and reduced critical print size compared with optical-only aids, although period and carryover effects necessitate cautious interpretation. Telerehabilitation trials showed functional gains comparable to conventional in-office programmes and were particularly effective for follow-up, reinforcement, and remote support. Quality-of-life-focused programmes achieved improvements in functional domains, whereas single-device interventions had a limited impact on overall quality-of-life outcomes. Nonrandomized studies contributed valuable contextual information on patterns of device utilisation, barriers to uptake, and pre-post functional improvements, though causal inference was limited.

Across studies, adults were most commonly affected by retinal and optic nerve disorders, while paediatric populations were primarily characterised by congenital or developmental visual impairments. Optical low-vision aids remained the most frequently prescribed interventions due to affordability and accessibility, whereas electronic devices offered superior functional performance but faced barriers to adoption. Functional outcomes showed moderate-to-large improvements in reading ability and speed, with gains in activities of daily living and independence, particularly when structured training was provided. Key barriers included device cost, limited awareness, cosmetic concerns, and inadequate follow-up. In terms of service delivery, telerehabilitation emerged as a feasible and effective alternative to in-office training, underscoring the importance of multidisciplinary teams and structured follow-up. Overall, evidence from randomised trials was relatively robust, while nonrandomized studies supported contextual understanding; the certainty of evidence was moderate for functional outcomes and low for quality-of-life outcomes.

Discussion

This systematic review synthesises available evidence on patterns of visual disability, utilisation of low-vision rehabilitation services, and prescription of low-vision aids, highlighting both clinical practices and service gaps across diverse settings. The findings underscore the critical role of rehabilitation in addressing functional limitations among individuals with irreversible vision loss.

Burden of Visual Disability and Need for Rehabilitation

Consistent with global estimates from the Global Burden of Disease (GBD) Study, visual impairment remains highly prevalent worldwide, particularly among older adults affected by age-related macular degeneration, diabetic retinopathy, and glaucoma [1,2]. These conditions frequently result in persistent functional impairment despite optimal medical or surgical care, necessitating comprehensive low-vision rehabilitation [3]. The substantial proportion of individuals with unaddressed vision impairment reported by the WHO further reinforces the importance of rehabilitation-focused interventions rather than curative strategies alone [5].

Patterns of Low-Vision Aid Prescription

Across included studies, optical magnification devices - such as handheld magnifiers, stand magnifiers, and telescopes - were the most commonly prescribed low-vision aids. Electronic devices, including closed-circuit televisions and digital magnifiers, were reported less frequently, often reflecting cost constraints and limited availability in resource-restricted settings. These patterns align with prior systematic reviews demonstrating variability in device prescription based on socioeconomic context and service infrastructure [27,28].

Functional Outcomes of Rehabilitation

Most studies reported improvements in task-specific functional outcomes, particularly reading ability, near work, and activities of daily living, following low-vision rehabilitation. Although outcome measures varied across studies, consistent gains in functional independence were observed, supporting the effectiveness of rehabilitation services when appropriately delivered [27,29]. However, heterogeneity in outcome assessment tools limited direct quantitative synthesis.

Barriers to Utilisation

Several studies highlighted barriers to effective utilisation of low-vision services, including a lack of awareness, delayed referral, limited access to trained professionals, and affordability of devices. These barriers were particularly pronounced in low- and middle-income regions, echoing concerns raised in WHO reports regarding inequitable access to vision rehabilitation services [3,5].

Overview of Evidence and Service Utilisation Patterns

The findings of this systematic review demonstrate substantial heterogeneity in the available evidence on low-vision service utilisation and low-vision aid (LVA) prescription patterns, particularly within the Indian context. Most included studies originated from tertiary care centres, introducing referral and access-related bias that may over-represent complex ocular conditions while under-representing rural and underserved populations. Consequently, population-level estimates of LVA uptake and utilisation may be underestimated, especially in peri-urban and rural settings where access to structured low-vision services remains limited [3,30,31]. Earlier community-based estimates may also under-represent reversible causes of visual impairment due to improvements in cataract and refractive error services over recent decades [1,30,31].

Clinical Presentation and Etiological Patterns

Across included studies, retinal degeneration, diabetic macular disease, and glaucomatous optic neuropathy predominated among adult low-vision populations, whereas pediatric cohorts more frequently presented with congenital ocular anomalies, albinism, corneal opacities, and cortical visual impairment. These trends are consistent with global epidemiological data on causes of visual impairment [1]. Functional improvement with LVAs was reported across diverse etiologies, supporting the role of rehabilitation irrespective of underlying diagnosis [8,24]. In age-related macular degeneration, optical LVAs such as high-add spectacles, hand magnifiers, and prisms improved near visual acuity and reduced activity limitation [9]. In corneal diseases, crossover trials demonstrated superior reading performance with video magnifiers compared to optical devices, likely due to improved contrast enhancement and glare control [10].

Optical Versus Electronic Low-Vision Aids

Traditional optical LVAs remain the most frequently prescribed and widely accepted devices owing to affordability, familiarity, and minimal maintenance requirements. However, electronic devices - including portable electronic vision enhancement systems (p-EVES), tablets, and closed-circuit television (CCTV) magnifiers - demonstrated superior task-specific performance, particularly for reading and near-vision activities, in selected populations [10,12,15]. Pediatric randomised trials showed tablet-based interventions to be feasible and effective in improving educational accessibility, highlighting the expanding role of digital LVAs in academic inclusion [11,12]. Portable electronic vision enhancement systems may be cost-effective for specific near-vision tasks but have limited impact on overall quality of life, supporting their role as adjuncts rather than replacements for conventional optical low-vision aids [15]. In patients with age-related macular degeneration, spectacle-based optical interventions demonstrated modest but task-specific improvements in visual performance, reinforcing the continued relevance of optical low-vision aids in routine rehabilitation practice [16]. While electronic magnifiers offered superior performance for certain near-vision tasks, optical magnifiers remained equally effective for many activities and were preferred by some users due to simplicity and ease of use [19]. Electronic vision enhancement systems provide significant improvements in reading speed and near-task performance compared with conventional optical aids, particularly for patients with severe visual impairment, supporting their role as effective adjuncts in low-vision rehabilitation [25].

Wearable Technologies

Wearable electronic vision enhancement systems represent an emerging domain in low-vision rehabilitation. Studies evaluating head-mounted displays reported high initial user enthusiasm and perceived benefits for reading, mobility, and leisure activities [13,20]. Nevertheless, practical limitations - including restricted field of view, image lag, device weight, and discomfort - were commonly reported and reduced long-term adoption [13]. A specialised wearable night-vision aid demonstrated meaningful expansion of functional visual fields in patients with concentric peripheral field loss, addressing a critical unmet need, although further refinement is required to enhance usability and real-world integration [26]. Structured self-management programs significantly improved vision-related quality of life and patient confidence, highlighting the importance of psychosocial and educational components in low-vision rehabilitation [18].

Telerehabilitation and Service Delivery Innovations

Telerehabilitation emerged as an effective alternative to in-clinic device training, with randomised trials demonstrating comparable improvements in reading performance and device proficiency between remote and in-person models [17]. High patient satisfaction and acceptability were consistently reported, particularly when personalised support was provided [14,20]. These findings are especially relevant for geographically dispersed populations and health systems with limited specialist availability, where telerehabilitation may reduce travel burden and device abandonment. Longitudinal analysis of low-vision clinic data revealed evolving service patterns with increasing emphasis on individualised device assessment and multimodal rehabilitation strategies tailored to patient needs [21].

Barriers to Utilisation

Persistent system-level barriers continue to limit broader adoption of low-vision services, including limited awareness among general ophthalmologists, delayed referrals, shortages of trained personnel, inadequate counselling, and lack of subsidy or insurance coverage [22,32]. Younger patients demonstrated particularly low awareness of assistive technologies, with non-availability cited as a major barrier [22]. These findings emphasise the need for policy-level interventions, improved procurement systems, and structured patient education programs.

Heterogeneity of Included Studies and Data Synthesis

Several included studies provided valuable contextual evidence but were not suitable for quantitative pooling due to heterogeneity in populations, interventions, and outcome domains. Pediatric and educational studies focused on feasibility and learning outcomes rather than standardised functional vision metrics [11,12]. Qualitative and feasibility studies offered insights into usability and patient experience but lacked extractable effect measures [13,20]. Observational service-based studies described real-world utilisation patterns rather than intervention efficacy [8,22], while self-management interventions addressed psychosocial outcomes through complex, multifaceted programs (not pooled). Studies evaluating specialised technologies assessed unique outcomes for which no comparable studies were available [15,26]. Collectively, this heterogeneity necessitated narrative synthesis rather than formal meta-analysis.

Strengths and Methodological Considerations

A key strength of this systematic review is the inclusion of methodologically diverse studies reflecting real-world low-vision rehabilitation practice. Although study designs, populations, and interventions varied, all addressed clinically relevant outcomes related to functional vision, quality of life, or device utilisation. Such diversity is appropriate for evaluating complex rehabilitation interventions, where randomised trials alone may not capture service delivery patterns, acceptability, and barriers to care [8,24].

Limitations and Future Directions

This review includes studies with heterogeneous designs, small sample sizes, varied outcome measures, and limited follow-up durations. Reliance on self-reported outcomes may introduce reporting bias. Future research should prioritise multicenter studies with standardised outcome measures, long-term evaluations of adherence and real-world device usage, cost-effectiveness analyses of low-cost indigenous electronic devices, and integrated service delivery models linking tertiary centres with district-level facilities.

Most of the included studies were conducted in tertiary-care settings, which may limit the generalizability of the findings to primary and community-based eye care. Nevertheless, the available evidence consistently supports the clinical value of low vision rehabilitation in improving functional vision, independence, and vision-related quality of life. Multidisciplinary rehabilitation teams play an essential role in patient assessment, selection and training in low vision aids, including visual field expansion devices, although their use may be limited by adaptation challenges and variable evidence. Driving-related difficulties in older adults with low vision further highlight the importance of comprehensive rehabilitation to enhance mobility and maintain independence. Although the feasibility of a meta-analysis was explored, substantial clinical and methodological heterogeneity across studies precluded quantitative synthesis, making a narrative approach more appropriate.

## Conclusions

Low-vision rehabilitation, using both optical and electronic devices, markedly improves functional outcomes for individuals with irreversible visual impairment. Globally, retinal diseases are the leading cause of adult visual disability, while congenital and developmental conditions predominate in children, reflecting diverse regional patterns. Optical LVAs remain the most widely used due to affordability and ease of use, whereas electronic devices and telerehabilitation enhance task-specific performance and access. A patient-centred, multimodal rehabilitation approach that integrates multiple device types, personalised training, and flexible delivery is essential to address unmet needs, promote adherence, and improve independence and quality of life among visually impaired populations worldwide.
